# Retraction Note: Enzyme mediated synthesis of hybrid polyedric gold nanoparticles

**DOI:** 10.1038/s41598-026-44642-x

**Published:** 2026-03-18

**Authors:** Célia Arib, Jolanda Spadavecchia, Marc Lamy de la Chapelle

**Affiliations:** 1 CNRS, UMR 7244, CSPBAT, Laboratoire de Chimie, Structures et Propriétés de Biomatériaux Et D’Agents Thérapeutiques Université Paris 13, Sorbonne Paris Cité, Bobigny, France; 2 Institut Des Molécules Et Matériaux du Mans (IMMM-UMR CNRS 6283), Le Mans Université, Avenue Olivier Messiaen, 72085 Le Mans Cedex 9, France

Retraction of: *Scientific Reports* 10.1038/s41598-021-81751-1, published online 05 February 2021

The Editors have retracted this Article at the recommendation of the National Centre for Scientific Research (CNRS) and University Sorbonne Paris Nord.

After publication of this Article and of subsequent Correction, concerns have been raised about the reported results, in particular that the size of the nanoparticles reported in the histograms in Figure 3 did not match TEM data included in the same figure. Throughout the process, the Authors were not able to provide the original data underlying this figure. An investigation conducted in parallel by the CNRS found that the data shown in both the original and the corrected versions of the Article appears to be physically unlikely. The Editors therefore no longer have confidence in the research presented in this work.

Célia Arib & Marc Lamy de la Chapelle did not explicitly state if they agree or disagree with the retraction. Jolanda Spadavecchia did not respond to the correspondence from the Editors about this retraction.

